# Facile simultaneous synthesis of tetraaniline nanostructures/silver nanoparticles as heterogeneous catalyst for the efficient catalytic reduction of 4-nitrophenol to 4-aminophenol†

**DOI:** 10.1039/d0ra03327h

**Published:** 2020-06-09

**Authors:** Sathish Mohan Botsa, Yarramsetti Pavan Kumar, Keloth Basavaiah

**Affiliations:** Dept of Inorganic & Analytical Chemistry, Andhra University Visakhapatnam India-530003 pavan.y525@gmail.com klbasu@gmail.com; National Centre for Polar and Ocean Research, Ministry of Earth Sciences Goa India-403804

## Abstract

Nanocomposites of tetraaniline (TAN) nanostructures/silver nanoparticles (Ag NPs) were synthesized by an interfacial polymerization method using *N*-phenyl-1, 4-phenylenediamine (NPPD), AgNO_3_ and ammonium persulphate (APS) as monomer, oxidizing agent in immiscible solvent toluene–water respectively. The structure and morphology of the as-prepared TAN and Ag NPs were investigated by UV-visible spectroscopy, Fourier transform infrared (FTIR) spectroscopy, X-ray diffraction (XRD), field emission scanning electron microscopy (FESEM), transmission electron microscopy (TEM), and thermogravimetry (TG). The results of FTIR spectroscopy confirmed the formation of TAN and Ag NPs and those of XRD showed the presence of the face centred cubic (fcc) phase of Ag NPs. The FESEM and TEM images gave direct evidence that Ag NPs stabilized with the TAN nanostructures. TGA indicated the enhanced thermal stability of the nanocomposites (NCs). The catalytic activity of TAN/Ag NCs was investigated for the model reduction of 4-nitrophenol (4-NP) to 4-aminophenol (4-AP) in the presence of excess sodium borohydride.

## Abstract 2

Nanocomposites of tetraaniline/silver nanoparticles were synthesised using an interfacial polymerisation method. The catalytic activity was investigated for the model reduction of 4-nitrophenol to 4-aminophenol in the presence of excess sodium borohydride.

## Graphical abstract

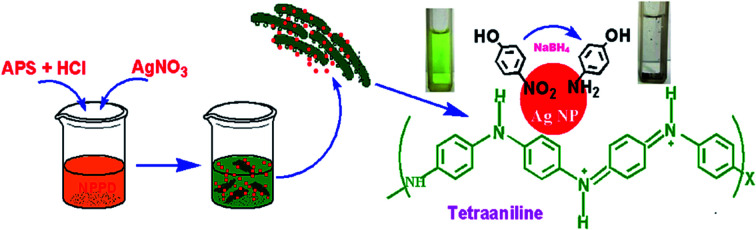

## 1. Introduction

Noble metal nanoparticles have received significant attention due to their numerous technological applications such as catalysis, medicine, electronics, and sensors.^1–3^ In particular, Ag nanoparticles have been studied because of their optoelectronic properties,^4–6^ which make them useful in numerous applications such as electronics, catalysis, and antimicrobial studies.^7,8^ To realize these applications, the size of the Ag nanoparticles must be below 100 nm but at this scale, the nanoparticles aggregate due to their high volume-to-surface ratio^9^ and result in irregular macrostructures, which can limit their applications. To overcome this hurdle, the surface of Ag NPs must be stabilized using surfactants, thiols, starch, polyacrylate, polyvinylpyrrolidone,^10–14^*etc.* Modifying agents or stabilizing agents also render these systems efficient for some applications. The immobilization of silver nanoparticles on different supports including polymers^15^ and inorganic supports such as metal oxides,^16^ carbon,^17^ and alumina^18^ can be an alternative choice for delivering better efficiencies and ease of separation from the reaction medium.^19^

Intrinsic conducting polymers (ICPs) are used in biosensors,^20^ gas sensors,^21^ and electromagnetic shielding devices.^22^ Copious conducting polymers (CPs) like polythiophene,^23^ polypyrrole,^24^ polyparaphenylene,^25^ and polyaniline^26^ are usually employed in various research areas. Among these, polyaniline (PANI) has been comprehensively studied due to its fascinating physical and chemical properties,^27^ convenient synthesis, low cost, and high thermal stability^28^ and its use in solar cells^29^ LEDs^30^ and energy storage devices.^31^ Recently, a new trend has evolved *i.e.* metal NPs have been introduced into the polymer matrix, resulting in hybrid polymer/metal NCs that are both polymeric and metallic in nature and deliver enhanced mechanical and thermal stability. In particular, PANI/Ag NCs have emerged as prominent materials due to their enhanced thermal, optical, mechanical, and conducting properties. In general, PANI/Ag NCs can be synthesized in two ways, *i.e.*, the simultaneous synthesis of PANI and Ag, where metallic salts are reduced by preformed polyaniline, and the synthesis of PANI in the presence of preformed Ag NPs. Among these two processes, the former gives much better homogenized PANI/Ag NCs.

PANI/Ag NCs in an ionic liquid were synthesized using one pot synthesis.^32^ PANI/Ag/polystyrenesulphonate (PSS) composites with particle sizes less than 20 nm were synthesized using the *in situ* polymerization method.^33^ PANI nanofiber/Ag composite networks were synthesized using a one-step method for antibacterial applications.^34^ PANI nanofibers with an average diameter of 20 nm and 5 nm Ag nanoparticles were simultaneously synthesized using a one-step method.^35^ PANI/Ag NCs were prepared *via* an *in situ* photo-redox reaction.^36^

Oligoanilines (OAs) can be an alternative to PANI as they have similar electronic, optical, and structural properties to PANI. Through the controlled synthesis of OAs, well-defined molecular structures, controlled chain lengths, and different redox states can be obtained. OAs have been synthesized *via* oxidative coupling, condensation, and palladium-catalyzed coupling.^37^ OAs, *i.e.*, tetramers and octamers were successfully prepared through oxidative coupling reactions in aqueous media.^30^ Structurally ordered TAN nanostructures such as 1-D nanowires, 2-D nanoribbons, and 3-D rectangular nanoplates and nanoflowers were prepared in the presence of a dopant acid by solvent exchange processes.^38^

Aromatic nitro compounds are widely used in pharmaceutical, pigment, dye, and pesticide industries. These compounds cause severe damage to humans, animals, and plants and the whole biological system when they are released into the environment. Among them, 4-nitrophenol (4-NP) is considered as a major organic pollutant by the US Environmental Protection Agency (EPA) as it has high solubility and stability in aqueous media and also because it gets accumulated on the soil surface and does not degrade. 4-NP is carcinogenic when inhaled into the human body. It affects the central nervous system, which results in hormone imbalance and slowly kills the working function of the kidney and liver; in addition, it irritates the eyes. In order to restrict this, we need an efficient method for the recovery of 4-NP. Several purification techniques such as advanced oxidation processes (AOPs), photocatalysis, UV irradiation, microwave radiation, and electrocatalysis have been reported for the purification of water contaminated with 4-NP, but all these methods need highly advanced instrumental techniques and no one method gives better results. However, the conversion of 4-NP to 4-aminophenol (4-AP) might give more satisfactory results than its removal from water. This is possible by the reduction of 4-NP to 4-AP with the aid of reducing agents such as hydrazine and lithium aluminium hydride (LiAlH_4_), but these are hazardous and also harmful to the environment. The complete reduction of 4-NP in presence of sodium borohydride (NaBH_4_) is not possible but can be achieved with the usage of metal nanoparticles with the help of NaBH_4_.

Therefore, we investigated the simultaneous synthesis of TAN/Ag NCs as a heterogeneous catalyst *via* the IP method. The formed tetraaniline (TAN) acted as a reducing agent, support, and stabilizer for the silver (Ag) nanoparticles. The prepared TAN/Ag NCs were further tested their catalytic activity on reduction of 4-nitrophenol (4-NP) to 4-aminophenol (4-AP).

## 2. Materials and methods

### 2.1 Chemicals

*N*-Phenyl-1,4-phenylenediamine (NPPD), silver nitrate (AgNO_3_), ammonium persulphate (APS), and nitric acid (HNO_3_) were purchased from Sigma Aldrich, India. 4-Nitrophenol (4-NP), sodium borohydride (NaBH_4_), and toluene were received from Merck Chemicals, India. Milli-Q water with a resistance greater than 18 MΩ was used for synthesis. All the chemicals were of analytical grade (AR) and were used without further purification.

### 2.2 Synthesis of TAN

In a typical synthesis process, a 0.01 M NPPD solution was prepared in water and then, 5 ml of 1 M HNO_3_ was added to 50 ml of an APS solution. Then, the acidified aqueous APS solution was added to the NPPD solution and the reaction was allowed to proceed at room temperature. After 30 minutes, the reaction mixture turned light green at the interface of toluene and water and gradually, the entire aqueous phase turned green, which indicated the formation of TAN. The reaction was allowed to proceed for 5 hours. The aqueous phase was then filtered and washed several times periodically with acetone and water and finally dried under vacuum at room temperature.

### 2.3 Synthesis of TAN/NCs

In a typical synthesis process, 0.01 M of 50 ml NPPD solution was prepared in toluene. Then, 20 ml of 1 M HNO_3_ was added to the NPPD solution. Next, 50 ml of 0.01 M AgNO_3_ solution and 50 ml of 0.01 M APS solution were prepared in Milli-Q water. The as-prepared 50 ml aqueous AgNO_3_ and APS solutions were added to 50 ml of the NPPD solution in a 250 ml beaker and then, the reaction was allowed to proceed at room temperature for 30 minutes. The aqueous phase of the reaction mixture turned light green and gradually turned dark green. The reaction proceeded for 12 hours. Then, the aqueous phase was separated and centrifuged. The supernatant liquid was discarded and periodical washing was done using acetone and water. Finally, the product was dried under vacuum at room temperature. A similar procedure was followed for the synthesis of TAN/Ag NCs by varying the molar ratio of monomer to AgNO_3_ as 1 : 0.125, 1 : 0.25, 1 : 0.5, and 1 : 1.

### 2.4 Characterization

UV-visible absorption spectra were recorded using a Shimadzu 2450 spectrophotometer. Fourier transform-infrared (FTIR) spectra were recorded over the range of 400–4000 cm^−1^ using a SHIMADZU-IR PRESTIGE-2 spectrometer. Powder samples were mixed thoroughly with KBr and pressed into thin pellets. Powder X-ray diffraction (XRD) patterns were recorded on a PANalytical X'Pert Pro diffractometer at a 0.02° s^−1^ scan rate using Cu-Kα radiation (45 kV, 40 mA). Transmission electron microscopy (TEM) images were obtained (TEM model FEI TECNAI G2 S-Twin) at accelerating voltages of 120 and 200 kV. Surface morphology was examined by scanning electron microscopy (SEM) using JEOL-JSM-6610 LV equipped with an electron probe-micro analyzer and FESEM (JSM-7500F). Thermogravimetric analysis (TGA) was carried out using a Shimadzu thermogravimetric analyzer (DTG-60H) with temperatures ranging from 25 to 800 °C under a stable N_2_ flow (50 ml min^−1^) and with a heating rate set at 10 °C min^−1^.

### 2.5 Investigation of the catalytic activity of TAN/Ag NC

To investigate the catalytic activity of TAN/Ag NCs, the reduction of 4-NP in the presence of NaBH_4_ was selected as a model reaction. In a typical reaction, a freshly prepared aqueous solution of NaBH_4_ (2.7 ml, 1 M) was added to an aqueous solution of 4-NP (1 ml, 7 × 10^−4^) in a quartz cell. Then, 1 mg of TAN/Ag NCs were added to the above solution. The reduction of 4-NP to 4-AP was monitored using a Shimadzu 2450 spectrophotometer at room temperature.

## 3. Results and discussion

**Fig. 1:**
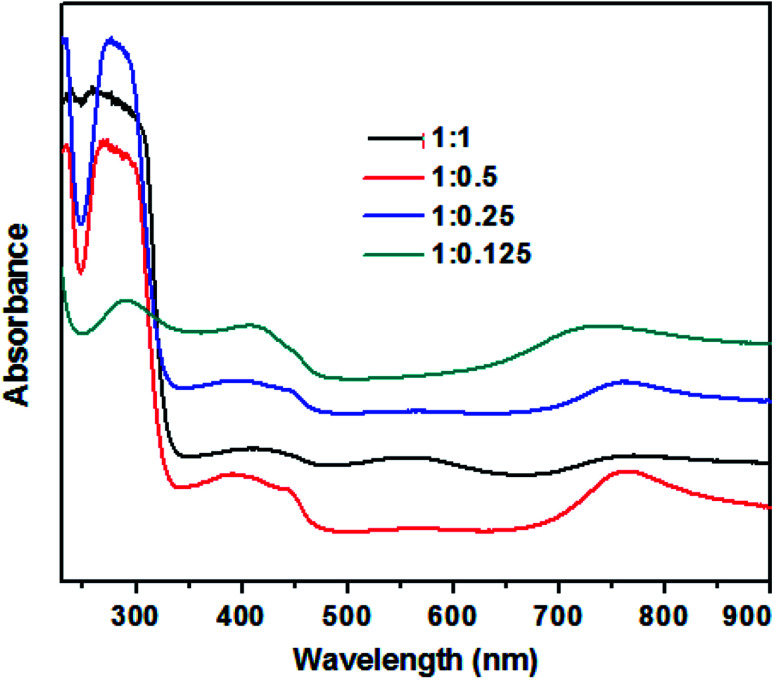
UV-visible absorption spectra of TAN/Ag NCs prepared at different molar ratios of NPPD to AgNO_3_.

The UV-visible absorption spectra of TAN/Ag NCs prepared at different molar ratios of NPPD to AgNO_3_ are depicted in Fig. 1. The three main characteristic absorption peaks can be observed at 290 nm, 410 nm and 720 nm. The absorption peak at 290 nm can be ascribed to the π–π* transitions of the benzenoid rings of TAN,^39^ while the peak at 720 nm is due to the polaron/bipolaron transitions and the extended conjugation of polymer rings.^40^ In addition, the peak at 560 nm (1 : 1 molar ratio) is due to the polaron–π* transition of the localized band and indicates the protonated/half-oxidized nature of TAN.^41^ In general, the surface plasma resonance (SPR) peak of Ag nanoparticles appears between 400 nm and 520 nm; this peak is sensitive to the particle size. Owing to high surface-to-volume ratio and great electron affinity to Ag nanoparticles in the composites can strip off the electron density from the surrounding TAN and thus, the electron density of the Ag nanoparticles increased resulting in a bathochromic shift of the SPR peak. The SPR peak of the Ag nanoparticles obtained at 410 nm confirmed that the Ag nanoparticles are embedded in TAN and it is overlapped by the polaronic peak of TAN.^42^

**Fig. 2:**
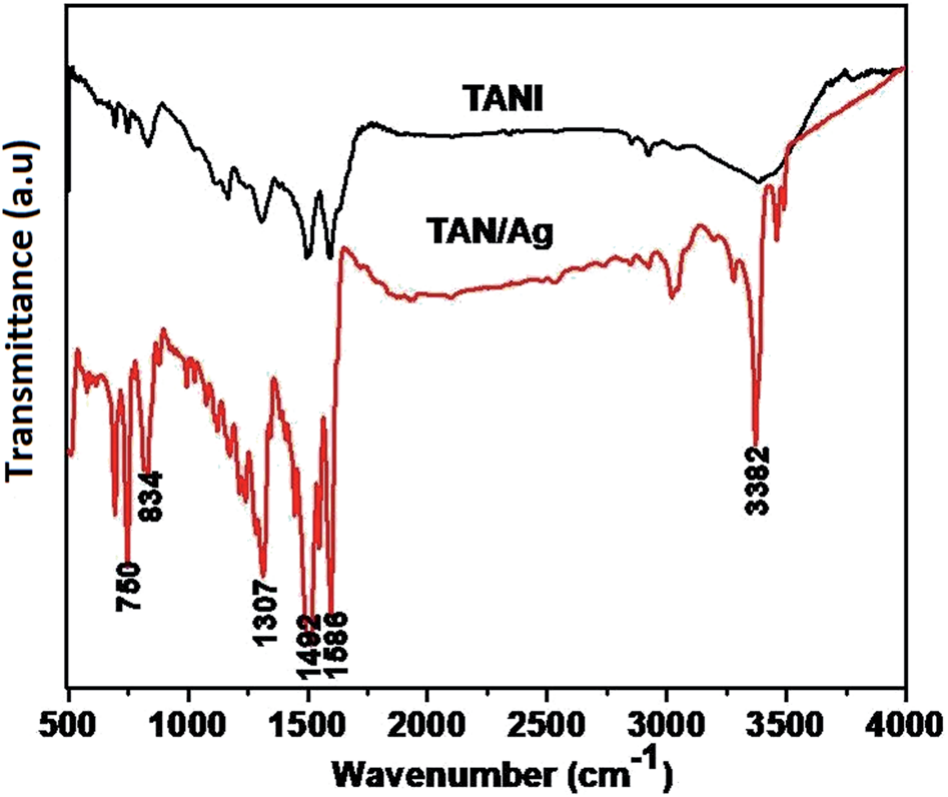
FTIR spectra of TAN and TAN/Ag NCs.

The FTIR spectra of TAN and TAN/Ag are presented in Fig. 2. The spectrum of TAN shows an absorption peak in the range of 550–750 cm^−1^, which is attributed to C–NO_3_^−^ bonding. The peak at 834 cm^−1^ is due to the N–H out-of-plane bending vibrations. The peak at 1163 cm^−1^ is because of the presence of the quinonoid ring (N=Q=N) and the peak at 1307 cm^−1^ is due to the C–N stretching mode vibrations. The peaks at 1492 cm^−1^ and 1586 cm^−1^ are due to the stretching vibrations of the benzenoid and quinonoid rings, respectively. The intensities of these two peaks are almost in a ratio of 1 : 1, which confirms that TAN is in the emeraldine base (EB) form. The other peak at 2919 cm^−1^ is due to the C–H stretching vibrations, whereas the peak at 3382 cm^−1^ is due to the N–H stretching vibrations.

The spectrum of TAN/Ag NCs shows absorption peaks at 692 cm^−1^ and 750 cm^−1^, which are ascribed to the C–NO_3_^−^ group. The C–H out-of-plane bending vibrations occur at 826 cm^−1^. The peak corresponding to C–N stretching vibrations can be observed at 1316 cm^−1^. The other two peaks at 1509 cm^−1^ and 1593 cm^−1^ are assigned to the stretching frequencies of the benzenoid and quinonoid rings, respectively.^43^ The peak at 3020 cm^−1^ is due to C–H absorption. The two peaks at 3365 cm^−1^ and 3458 cm^−1^ can be assigned to the N–H stretching vibrations. The other two peaks at 1121 cm^−1^ and 1240 cm^−1^ are due to the C–C stretching and C–C twisting frequencies of the alkyl chain. As mentioned above, there is a shift in the frequencies of the peaks of TAN and TAN/Ag NCs, which confirms the interaction between Ag and TAN.

**Fig. 3:**
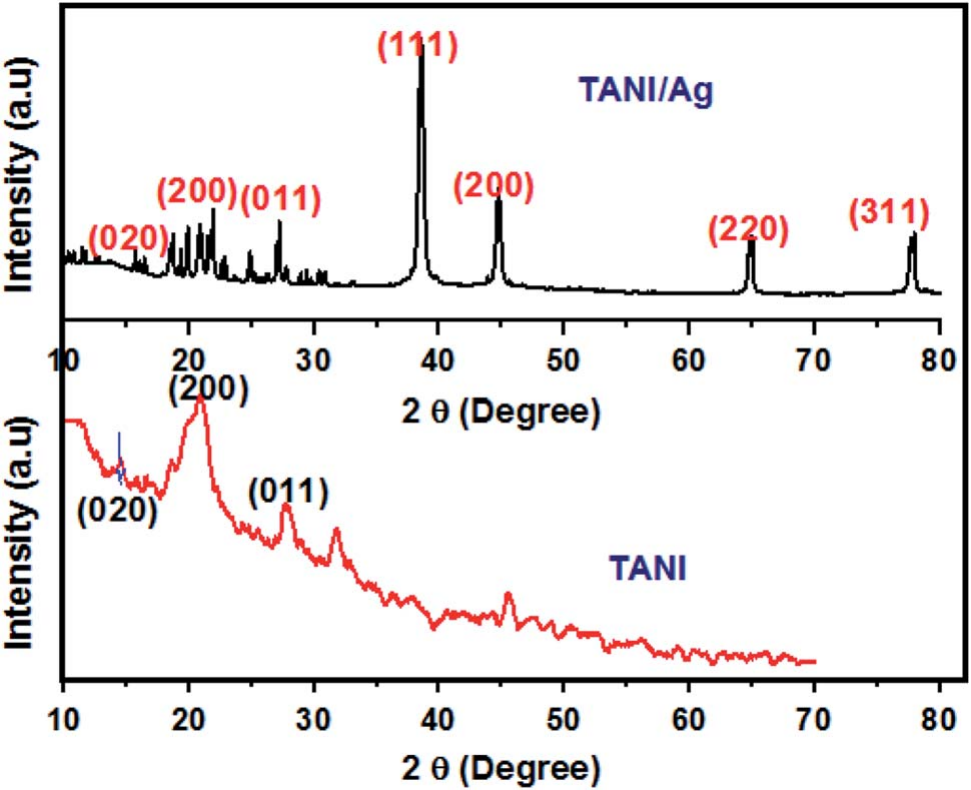
XRD patterns of TAN and TAN/Ag NCs.

The XRD patterns of TAN/Ag NCs (molar ratio, 1 : 0.125) and TAN are presented in Fig. 3. The XRD patterns of TAN displays two major peaks at 2*θ* = 20.7° and 28.2° contributes to the periodicity parallel and perpendicular to the phenyl ring of TAN which confirmed the formation of the (110) plane. In addition, another peak at 2*θ* = 14.6° indicates the amorphous nature of TAN. Besides the TAN features, four peaks are observed for TAN/Ag NCs with the 2*θ* values of 38.4°, 44.9°, 64.9° and 77.9°. These peaks represent Bragg reflections from the (111), (200), (220), and (311) planes of a face centered cubic lattice. The peak at 2*θ* = 38° is the characteristic peak of Ag NPs. The existence of sharp peaks clearly indicates the presence of Ag NPs in the TAN matrix and their crystalline nature. The average particle size of Ag NPs in NCs was calculated for the most intense peak at the (111) Bragg reflection using the Debye–Scherrer equation^43^ and it was found to be 22 nm.

**Fig. 4:**
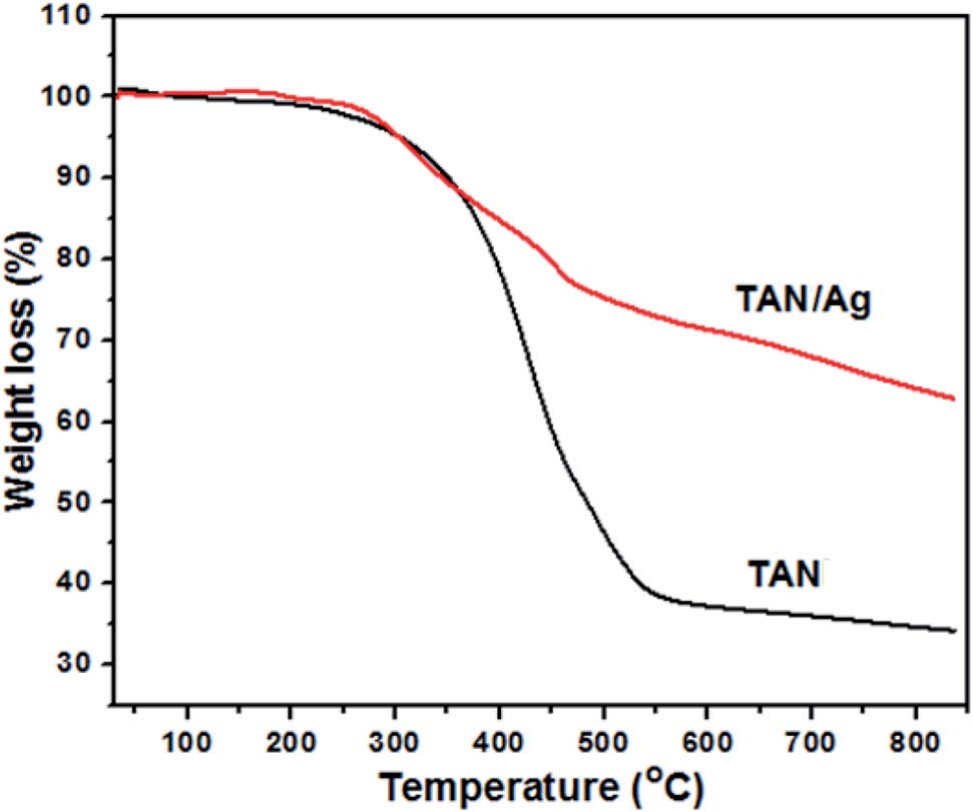
Thermograms of TAN and TAN/Ag NCs.

The thermograms of TAN/Ag NCs and TAN under an N_2_ atmosphere with a heating rate of 20 °C min^−1^ in the range of 30–840 °C are shown in Fig. 4. It was observed that TAN and TAN/Ag NCs underwent three major steps of weight loss. The first small fraction of weight loss in the temperature range from 30 to 270 °C was due to the loss of water molecules present at the surface of TAN and TAN/Ag NCs held through weak ionic interactions.^27^ TAN showed 3.2% weight loss, whereas TAN/Ag NCs showed only 0.5% weight loss in the first step. The small weight loss (11.1%) in the range of 270–375 °C was due to the volatilization and loss of nitrate dopant ions (NO_3_^−^) in TAN. The second major step of continuous weight loss occurred in the temperature range of 270–540 °C and could be assigned to the vanished oligomers in TAN (48.3%) and TAN/Ag NCs (23.1%).^44^ In the third step of the decomposition process, gradual weight loss was observed due to the thermal oxidative decomposition and degradation of the polymer fraction of NCs.^45^ TAN showed 4.7% weight loss, whereas TAN/Ag NCs showed 14.1% weight loss. From the graph, it can be seen that NCs have greater thermal stability than TAN probably due to the presence of Ag NPs.^46^

**Fig. 5:**
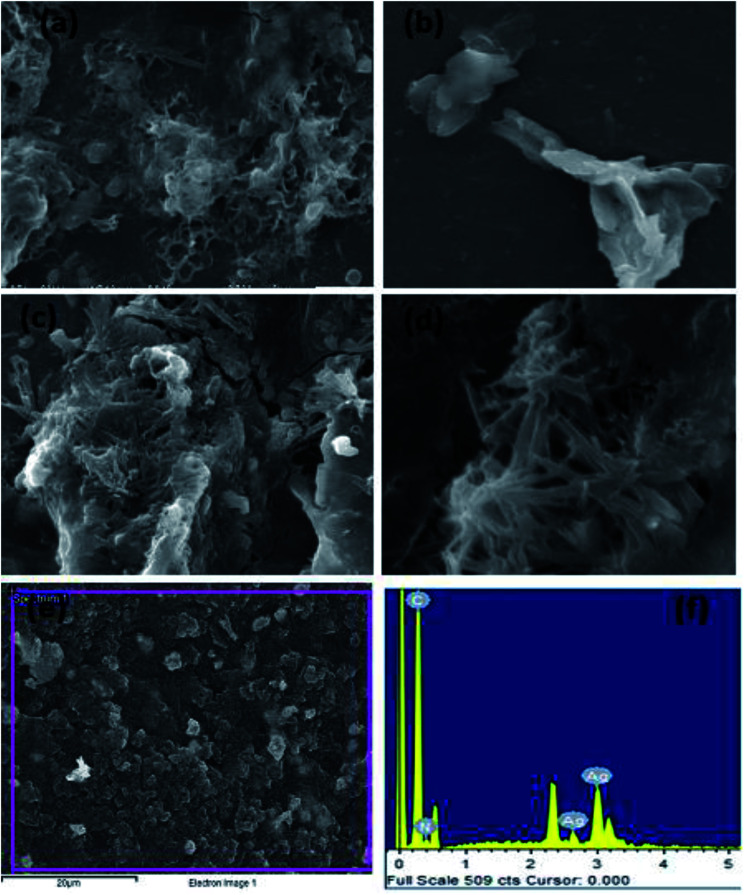
SEM images of TAN/Ag NCs at different molar ratios of NPPD to AgNO_3_ (a) 1 : 1, (b) 1 : 0.5, (c) 1 : 0.25, (d) 1 : 0.125, (e) and (f) EDX of TAN/Ag NCs.

**Fig. 6:**
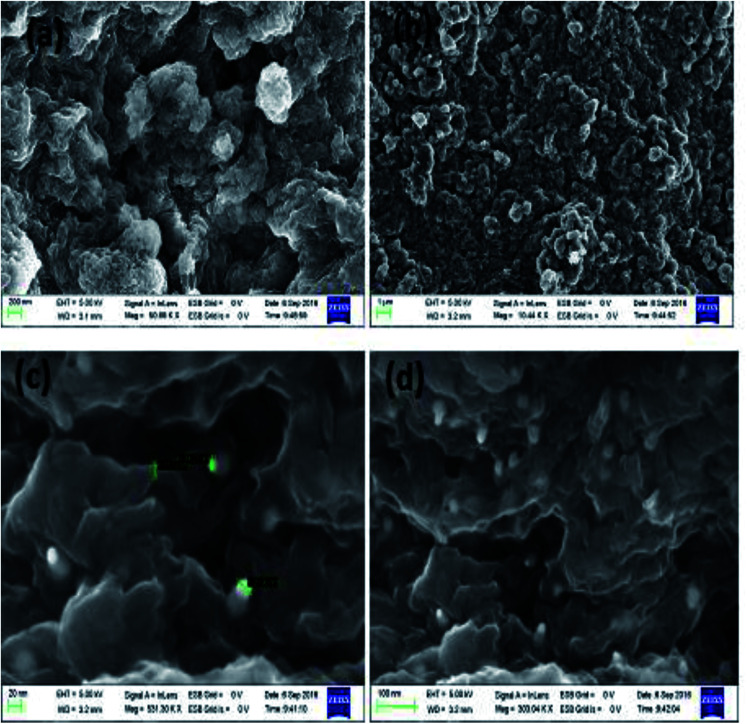
FESEM images of TAN (a and b) and TAN/Ag NCs (c and d).

Morphological studies of the synthesized TAN and TAN/Ag NCs were conducted using scanning electron microscopy (SEM). From the SEM images shown in Fig. 5, we can infer that the molar ratio of NPPD to AgNO_3_ has a profound effect on morphologies. 1-D nanorods were obtained for TAN/Ag NCs as the molar ratios of NPPD to AgNO_3_ decreased from 1 : 1 to 1 : 0.125. Higher molar ratios of NPPD to AgNO_3_ (1 : 1, 1 : 0.5) may fracture the formed TAN rings due to the higher oxidation by AgNO_3_, resulting in plate-like structures. The energy dispersive X-ray (EDX) spectroscopy results of TAN/Ag NCs (1 : 0.125) confirmed the presence of elements such as C, N, and Ag (Fig. 5(e) and (f)). Fig. 6(a–d) show the FESEM images of TAN and TAN/Ag NCs (1 : 0.25), respectively. The FESEM images of TAN/Ag NCs confirmed that Ag NPs were spherical in shape and embedded in the TAN matrix.

**Fig. 7:**
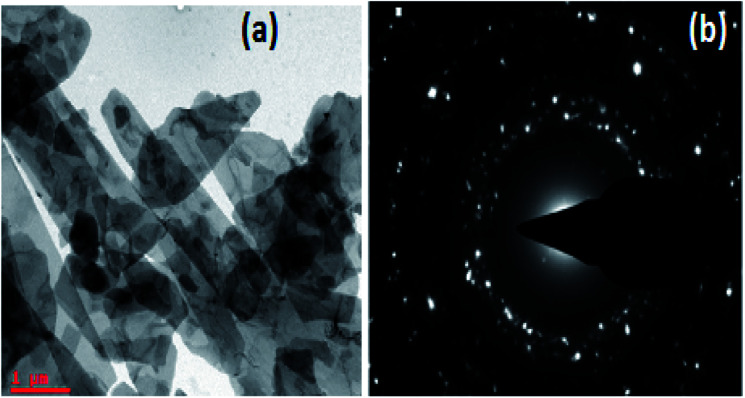
(a) TEM image of TAN/Ag NCs and (b) the SAED pattern of TAN/Ag NCs.

The TEM image of TAN/Ag NCs presented in Fig. 7(a) shows that the lengths of the TAN rods are in the range of several micrometers and Ag NPs are embedded in the TAN matrix. Fig. 7(b) exhibits the selected area electron diffraction (SAED) pattern of TAN/Ag NCs, which matches with the planes of XRD.

### 3.1 Formation mechanism of TAN/Ag NCs

**Fig. 8:**
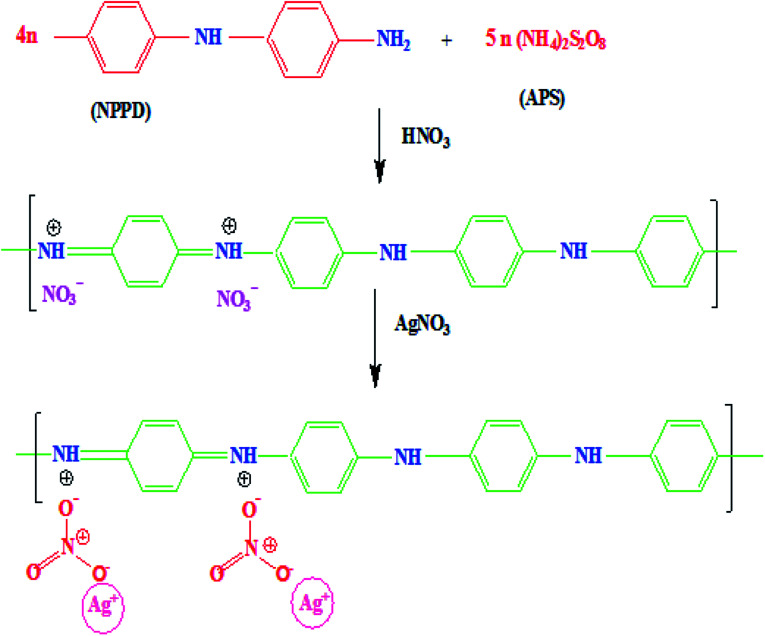
Schematic representation of the formation of TAN/Ag NCs.

The monomer NPPD first reacted with HNO_3_ and produced protonated ions in toluene; these ions polymerized when an aqueous APS solution was slowly added to the toluene solution. The polymerization process begins at the interface of toluene and water. The ions aggregated together and extended into straight chains (1, 4 joining of phenyl groups) but side-products were not observed due to the fast reaction of anilinium ions. Protonation occurred on the imine unit of the quinonoid part of EB TAN and the lone pair of electrons on nitrogen was removed to generate a positive charge. The free NO_3_^−^ accumulated on EB TAN (green colour) because of the electrostatic attraction between NO_3_^−^ and TAN (Fig. 8). The metallic salt solution (AgNO_3_) was finally reduced to Ag^0^ by the preformed TAN through the diminished charge of Ag^+^ ions, which interacted with the counter ions of NO_3_^−^, as presented in Fig. 8.

### 3.2 Evaluation of the catalytic activity of TAN/Ag NCs

**Fig. 9:**
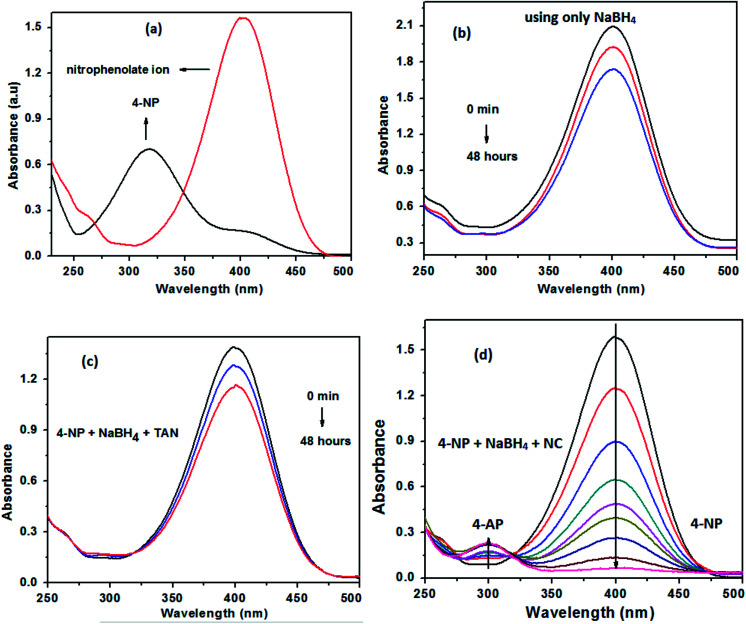
(a) Formation of the nitrophenolate ion, (b) reduction of 4-NP with only NaBH_4_, (c) reduction of 4-NP in the presence of NaBH_4_ and TAN and (d) the reduction of 4-NP with TAN/Ag NCs in the presence of NaBH_4_.

The reduction of 4-nitrophenol (4-NP) using TAN/Ag NCs in the presence of NaBH_4_ was chosen as a model reaction for the evaluation of the catalytic activity of NCs. The reduction of 4-NP was carried out at room temperature (25 °C) in a standard quartz cell having 1 cm path length and monitored by UV-visible spectroscopy. Only 4-NP showed an absorption peak at 317 nm. The addition of NaBH_4_ to 4-NP gives 4-nitrophenolate ions (yellow colour), which results in a significant bathochromic shift from 317 nm to 400 nm (Fig. 9(a)); this is due to the auxochromic nature of 4-NP. However, the complete reduction of nitrophenolate ions did not occur at 400 nm with either NaBH_4_ or TAN in the presence of NaBH_4_ even when the solution was kept for 48 hours (Fig. 9(b and c)). Normally, the reduction of 4-NP to 4-AP using aqueous NaBH_4_ is favourable; however, this reaction could not progress due to the large potential difference between the donor and acceptor molecules. When TAN/Ag NCs were added to the mixture of NaBH_4_ and 4-NP solutions, Ag NPs catalyzed the reaction by providing electrons from the donor BH_4_^−^ to the acceptor 4-NP. The colour change in the solution as well as the reduction process was monitored every 2 minutes. The intensity of the 4-nitrophenolate ion peak at 400 nm was found to decrease with time and simultaneously, a new peak appeared at 300 nm, which indicated the formation of 4-aminophenol (4-AP), as shown in Fig. 9(d). However, initially, 4-NP lies on the surface of TAN/Ag NCs and then, reduction occurs. The ability to reduce 4-NP to 4-AP increases through the adsorption process.^47^ The reduction process was assumed to be a pseudo-first-order reaction since the concentration of NaBH_4_ greatly exceeded that of 4-NP. A straight line was obtained by plotting the graph of “ln *C*_*t*_/*C*_0_” *vs.* time. The rate constant values of the reduction process were determined by measuring the intensity of the nitrophenolate ion peak at 400 nm as a function of time.

#### 3.2.1 Effect of different TAN/Ag NCs on the reduction of 4-AP

**Fig. 10:**
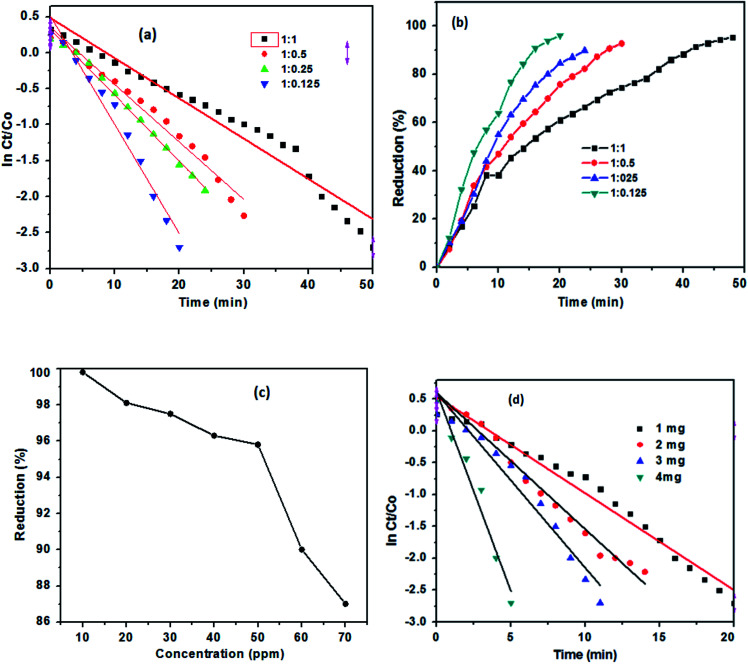
(a) ln *C*_*t*_/*C*_0_ plots for different molar ratios of TAN/Ag NCs prepared with NPPD and AgNO_3_ and (b) percentage of the reduction of 4-NP with different TAN/Ag NCs. (c) Effect of 4-NP concentration on the reduction of 4-NP and (d) ln *C*_*t*_/*C*_0_ plots for different weights of TAN/Ag NCs (1 : 0.125).

The catalytic activity of TAN/Ag NCs prepared at four different molar ratios of NPPD to AgNO_3_ (1 : 1, 1 : 0.5, 1 : 0.25, and 1 : 0.125) was investigated against 4-NP. The prepared samples such as molar ratios of 1 : 1, 1 : 0.5, 1 : 0.25, and 1 : 0.125 were completed the reduction process in 50 (maximum), 30, 24, and 20 minutes, respectively (Fig. 10(a)). The effective reduction time for different TAN/Ag NCs varied; this might be due to the different morphologies at different molar ratios of NPPD to AgNO_3_.^48^ The percentage of reduction for TAN/Ag (1 : 0.125) was 97%, while those for TAN/Ag NCs were 92%, 90% and 96% for 1 : 0.25, 1 : 0.5, and 1 : 1 molar ratios of NPPD to AgNO_3_, respectively, as shown in Fig. 10(b). These results are presented in Table 1. Among tested samples, the TAN/Ag NCs with a molar ratio of 1 : 0.125 exhibited the maximum conversion rate of 4-NP to 4-AP because a higher molar ratio of NPPD to AgNO_3_ (1 : 1, 1 : 0.5) may fracture the formed TAN rings due to increased oxidation by AgNO_3_, as discussed in the morphology study. This would overload the catalyst loading which resulting in no more active sites for the adsorption of 4-NP on the surface of catalyst.

**Table 1:** Effect of different TAN/Ag NCs with different molar ratios of NPPD to AgNO_3_ on the reduction of 4-NP

TAN/Ag NCs	Time (min)	Reduction (%)
1 : 1	50	90
1 : 0.5	30	92
1 : 0.25	24	96
1 : 0.125	20	99

#### 3.2.2 Effect of 4-NP concentration on the reduction of 4-NP

To investigate the effect of the concentration of 4-NP on the reduction process, initially, 10 ppm of 4-NP in the presence of NaBH_4_ was tested with 1 mg of TAN/Ag NCs (1 : 0.125) and keeping the amount of catalyst loading amount constantly but changed in the different concentrations of 4-NP, the maximum reduction rate (99%) was noticed in Fig. 10(c). Upto 50 ppm of 4-NP solution, the reduction process takes place at a higher rate and thereafter, the reduction process consumes more time though the complete reduction is not achieved.

#### 3.2.3 Effect of TAN/Ag NC dosage on the reduction of 4-NP

The effect of TAN/Ag NCs dose (molar ratio 1 : 0.125) were further examined with 1 mg, 2 mg, 3 mg and 4 mg of catalyst on the reduction of 4-NP is presented in Fig. 10(d). The results stated that increasing in catalyst amount tend to reduced time for the reduction of 4-NP. When 4 mg of catalyst load used, the reduction time was taken in 6 minutes only and it is contrast to 3 mg, 2 mg and 1 mg of catalyst dose, their reduction times were reported at 12, 14, and 20 minutes respectively. The rate constant values for the catalytic reduction of 4-NP to 4-AP are given in Table 2.

**Table 2:** Effect of the dosage of TAN/Ag NCs on the reduction of 4-NP

TAN/Ag NCs (1 : 0.125) (mg)	Time (min)	Rate constant (min^−1^)
1	20	15.2 × 10^−2^
2	14	21.4 × 10^−2^
3	12	27.4 × 10^−2^
4	6	62.6 × 10^−2^

#### 3.2.4 Reusability of TAN/Ag NCs

**Fig. 11:**
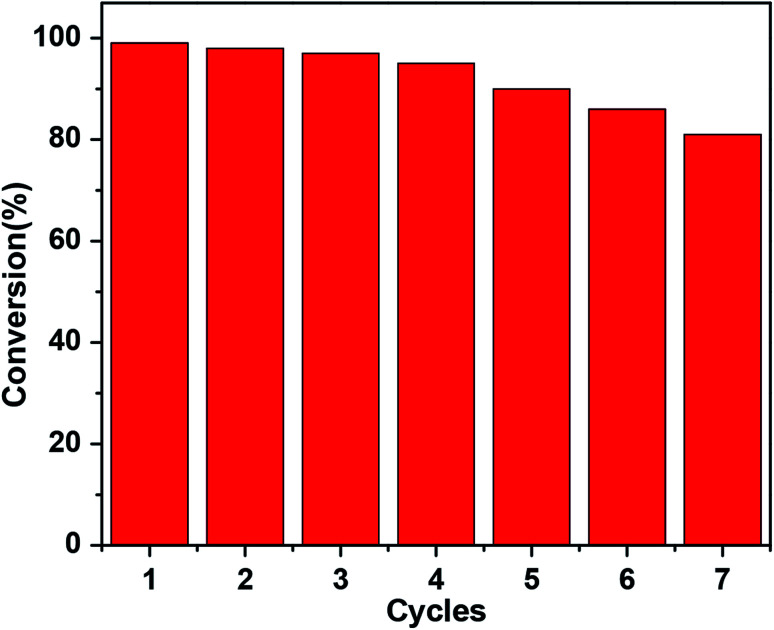
Recyclability of TAN/Ag NCs for the reduction of 4-NP.

The reusability and stability of a catalyst are of great importance for its practical applications. The current catalysts are easily recoverable from the reaction mixture after the completion of the 4-NP reduction to 4-AP with NaBH_4_. In order to assess the reusability and sustainability of the current catalysts, successive catalytic cycles with the catalysts were carried out. The reusability of TAN/Ag NCs is presented in Fig. 11; it indicates that up to the 5^th^ cycle, the conversion of 4-NP to 4-AP is above 90%. Thereafter, the conversion decreases to 86% for the 6^th^ cycle and 81% for the 7^th^ cycle. This decrease in conversion can be attributed to the loss of the catalyst during the filtration of the catalyst from the reaction mixture.

### 3.3 Reduction mechanism of 4-NP

**Fig. 12:**
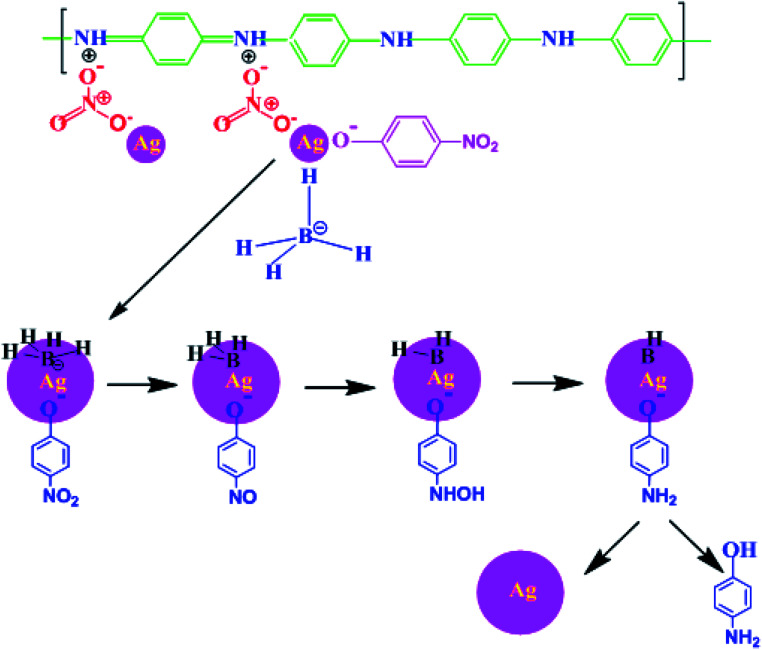
Schematic representation of the reduction of 4-NP to 4-AP using TAN/Ag in the presence of NaBH_4_.

After adding TAN/Ag NCs to the solution, both BH_4_^−^ and nitrophenolate ions chemisorbed onto the surface of the catalyst. The functional group NO_2_ in the nitrophenolate ions was converted to NO with the loss of OH^−^ ions.^49^ The addition of water occurred on the catalyst; NO was converted to NHOH and finally to OH, as presented in Fig. 12. Successive washings of the surface of the catalyst with water resulted in free 4-AP.

## 4. Conclusions

In this work, a simple and effective approach was developed to obtain TAN and TAN/Ag NCs using the IP method. The UV-vis spectra showed that the electron density on Ag NPs resulted in a bathochromic shift in the SPR peak of Ag NPs. The thermograms of TAN/Ag NCs and TAN revealed that TAN and TAN/Ag NCs underwent three steps of weight loss. The XRD pattern of TAN indicated four peaks for TAN/Ag NCs with the 2*θ* values of 38.4°, 44.9°, 64.9°, and 77.9°. The SEM images suggested that the morphology of TAN/Ag NCs could be tuned with respect to the molar ratio of NPPD to AgNO_3_. 1-D nanorods were obtained at a molar ratio of 1 : 0.125 (NPPD to AgNO_3_). The reduction of 4-NP using TAN/Ag NCs in the presence of NaBH_4_ was optimized and chosen as a model reaction for the evaluation of the catalytic activity of NCs.

## Conflicts of interest

The authors have no conflicts of interest to declare.

## Supplementary Material

RA-010-D0RA03327H-s001
