# Supplementary material for: Facile simultaneous synthesis of tetraaniline nanostructures/silver nanoparticles as heterogeneous catalyst for the efficient catalytic reduction of 4-nitrophenol to 4-aminophenol
Source: RSC Adv. 2020 Jun 9;10(37):22043–53. doi: 10.1039/d0ra03327h (PMC9054504; doi:10.1039/d0ra03327h)
Supplement: RA-010-D0RA03327H-s001 [file RA-010-D0RA03327H-s001.pdf]

Electronic Supplementary Material (ESI) for RSC Advances.
This journal is © The Royal Society of Chemistry 2020

Supporting Information

Table S1: Kinetic rate constants of different molar ratios of TAN/Ag NC prepared with NPPD to $AgNO_3$

Time (min)	1:1	1:0.5	1:0.25	1:0.125
0	0.26005	0.53493	0.26005	0.45616
10	0.19451	0.35331	0.14583	-0.1087
20	0.14583	0.25813	0.0198	-0.43696
30	0.11532	0.10803	-0.1087	-0.92382
40	-0.1087	-0.11207	-0.3581	-1.9951
50	-0.2145	-0.49457	-0.55165	-2.70306
